# Ostracism affects children’s behavioral reactivity and gaze cueing of attention

**DOI:** 10.1371/journal.pone.0320338

**Published:** 2025-03-26

**Authors:** Giada Basset, Alessia Testa, Chiara Turati, Ermanno Quadrelli, Hermann Bulf

**Affiliations:** 1 Department of Psychology, University of Milano-Bicocca, Milan, Italy; 2 NeuroMI, Milan Center for Neuroscience, Milan, Italy; NTT Communication Science Laboratories, JAPAN

## Abstract

Being ostracized is a negative experience that threatens important psychological needs, inducing considerable cognitive and behavioral changes and influencing the processing of social signals such as gaze-cueing. Yet, little is known about how self-experienced ostracism affects children’s behavior and attentional processes. The present study aims to explore whether the social experience of being included or ostracized can modulate gaze-cueing of attention and behavioral reactivity in 6- (N = 40) and 10-year-old children (N = 40) and adults (N = 50). Participants were video-recorded while playing an online ball-tossing game (i.e., Cyberball), where they could be either included or ostracized. They then participated in a gaze cueing task, where the cue was provided by the eye-gaze of a central human face, and the target could appear in a congruent or incongruent position. Results revealed that ostracism affected both adults’ and children’s ability to follow another’s gaze, as they were slower to respond to incongruent targets when ostracized compared to when included. Additionally, ostracism impaired 10-year-old children’s accuracy in responding to the target. Behavioral reactivity results demonstrated that both children and adults were more disappointed during the ostracism vs. inclusion condition. Overall, current findings demonstrate that self-experienced ostracism modulates children’s and adults’ behavioral reactivity and processing of social signals such as gaze cueing.

## Introduction

Ostracism, the painful experience of being ignored and kept apart from others [1,2], is a form of social exclusion [3] that people can experience in their daily lives across the lifespan [4,5]. From early developmental stages [6], individuals desire to form social attachments and resist losing them, even when the maintenance of the bond is challenging [7]. Furthermore, being ostracized threatens the four fundamental human needs [2] of belonging [7], control over the interactions with others [8], meaningful existence [9], and self-esteem [10]. An episode of ostracism can cause behavioral and cognitive changes that people implement to react to social exclusion [11], such as behaving in appealing ways to others [12] and deploying attentional resources to manage the distress induced by the experience of being left out [13].

Ostracism is known to impact on the processing of social stimuli, such as emotional faces and eye gaze [14–17], which are essential sources of socially relevant information and affect our ability to interact efficiently with others [18]. These results showed that being left out motivates social reconnection with others, affecting basic perceptual and attentional processes and not only explicit and conscious choices. Recently, Bossi and colleagues [14] investigated whether ostracism affects adults’ accuracy in identifying the direction of gaze on human faces, which is a relevant social cue that can communicate to us where the other person is looking and what is attending [19,20]. Adult participants took part in the Cyberball paradigm, a well-known computer game where a ball is usually passed between three players, one of whom is the participant and the other two are computer-controlled players [12]. Participants can be included (i.e., each player receives and tosses the ball equally) or ostracized (i.e., after a few throws at the beginning, the participant no longer receives the ball). Ostracized participants were less accurate in identifying the gaze direction of human faces than included ones. The authors interpreted these results as the consequence of a social cognition impairment [14], which affected the tendency to allocate attention to potential re-inclusive social signals after experiencing an ostracism episode, as in the case of eye gaze. Furthermore, after being ostracized from a Cyberball game, adults’ cueing effect was affected in the presence of a human gaze but not for non-social stimuli like an arrow, demonstrating that the impact of ostracism on the cueing effect is specific for social rather than non-social stimuli [15]. More recently, Yang and colleagues [21] also underlined that social exclusion makes individuals less likely to follow the gaze of those who excluded them, further supporting the hypothesis that the perception of social relationships can influence how adults process social signals. Overall, these studies indicate that social inclusion vs. ostracism can affect adults’ processing of social stimuli, such as eye gaze.

Beyond the studies mentioned above focusing on adults, there has also been significant research aimed at exploring the multilevel and multidimensional effects of ostracism across childhood. Indeed, it has been shown that experiences of social exclusion significantly impact children’s behavioral and cognitive processes, even at early stages of development [17,22,23]. For example, at 5 years of age, ostracized children exhibited more affiliative behaviors, such as increased imitation of others’ actions or language choices, compared to those who were included [22,23]. Developmental studies echo findings from adult research, demonstrating that ostracism threatens fundamental needs in adolescents and children as well [24]. Moreover, children’s cognitive performance is poorer after being ostracized [25] and children’s logical reasoning appears worse [26]. Research also highlighted developmental differences in ostracism’s influence on cognitive and behavioral processes. For example, unlike 10-year-old children, 5-year-olds’ ability to recognize emotional faces is affected by an ostracism episode [17]. Furthermore, affective and regulatory brain regions crucial for social processing (ventrolateral prefrontal cortex and ventral anterior cingulate cortex) show increased activation and connectivity during an ostracism event from childhood to adolescence [27]. These areas, in which the major peak of gray matter volume is reached around 11–12-years of age [28], are known to be linked with the neural processing of social exclusion [27]. Indeed, the activation of the ventrolateral prefrontal cortex was mainly observed with developmental samples when compared to adults [29], suggesting that the brain’s response to social exclusion changes across development.

Despite the increasing research interest in the effects of social exclusion throughout development, no study, to the best of our knowledge, has yet investigated whether ostracism can modulate the ability to follow another’s gaze during the school-age periods. However, investigating this phenomenon in children would allow us to explore whether the results reported in adult literature [14,15] are generalizable to the developmental population or if there are unique developmental effects to consider. Given the established link between gaze-following, language and social development [30,31], it is crucial to investigate whether ostracism affects gaze-following in school-aged children. Our ability to interact effectively with others strongly depends on our ability to attend to social signals from an early age [18]. Therefore, understanding how ostracism impacts gaze-following can provide valuable insights into its potential consequences for children’s cognitive and affective development. Privileged attention towards human face, eye contact and gaze following have been observed since the first months of life [32–36] and have been proven to have cascading effects on the emergence of subsequent developmental competences in several domains. For example, they are associated with the early manifestation of joint attention [37] and the development of social communication [38], and may differentiate typically developing children from children with atypical development and socio-communicative difficulties [36].

In addition, the eyes play a very important role in social interactions, as being able to interpret the gaze of others can reveal what another person is looking at and what is attending to [36]. Eye-gaze following was studied across development using attentional tasks in which a peripheral location is cued by an eye-gaze displayed by a schematic or human face, both during real interactions and with computerized tasks inspired by the Posner task [36,39,40]. This task is typically administered by showing a central cue-stimulus which signals a peripheral location. A peripheral target can then appear either in the same or opposite location of the one signaled by the cue. In a gaze-cueing task, Swettenham and colleagues [36] found that 8- to 11-year-old children were faster at detecting a target when it appeared in a congruent position with a preceding eye-gaze cue compared to when it appeared in an incongruent position. In line with this evidence, it was shown that, during a Posner-like cueing task, 11-year-old children’s attention was more affected by the direction of eye gaze than by an arrow [34]. Additionally, the age of the person (i.e., a child or an adult) giving the cue was found to be irrelevant for the cueing effect, which remains present in 6- to 14-year-old children [41]. Given the early emergence of gaze following [33], its relevance for the development of social competences [38] and its modulation in response to ostracism in adults [14,15], it is surprising that the effects of ostracism on the gaze-cueing effect in children have not yet been investigated. In addition, investigating the effects of being left out on gaze-following across development appears crucial, as it has been shown that, while basic attentional mechanisms are present in young children, gaze evolves into a social cue with distinct attentional properties over time, with full development likely not occurring until adolescence [42]. Therefore, not only ostracism may be processed differently across age groups, but gaze-following may also be affected in varying ways across development.

The aim of the present study was to explore whether being included or ostracized can influence the processing of gaze direction in 6-, 10-year-old children and adults. After being included or ostracized in a Cyberball game [12], participants were presented with a questionnaire to assess their fundamental needs threat and, subsequently, with a gaze cueing task in which they were required to detect a target appearing on the right or left of a central face orienting the eyes either to the left or right. Developmental differences in behavioral reactivity during the Cyberball game were also evaluated. We chose to maintain the same task and dependent variables across all three age groups considered. For this reason, we decided to include 6-year-old children rather than younger ones, ensuring that they had the ability to understand the task and instructions, allowing us to record not only response accuracy but also reaction times. Additionally, we selected two age groups at the beginning and end of the same educational cycle, primary school, to keep this variable constant. In line with previous studies, we expect that both ostracized adults and children will report higher threat of fundamental needs and feelings of being ignored in the questionnaire [24] (Hypothesis 1). In addition, and as demonstrated by previous studies [23,43,44], we expect that children and adults will express more smiles and involvement (expressed by leaning forward and bending head on the side) and less distress behavior and disappointment when included at the Cyberball game (Hypothesis 2). This measure will also serve as an implicit index of perceived exclusion in both children and adults, potentially complementing the results obtained from the questionnaire. Given that younger participants may face difficulties in accurately reporting their feelings of exclusion due to questionnaire age-related factors [45], this behavioral reactivity analysis could provide valuable additional insights. Furthermore, in line with adult literature [14,15], for the gaze cueing task we expected that the facilitation provided by gaze direction would be reduced in ostracized children and adults as compared to included participants. Therefore, we can expect higher accuracies and shorter reaction times for the included participants, which will show a greater gaze-cueing effect as compared to ostracized participants [15] (Hypothesis 3a). Alternatively, as individuals are slower to disengage from social stimuli after being left out [16], another hypothesis could be that our participants will be more engaged by eye gaze after being ostracized, thus having difficulties and being slower to disengage from the central face. In this second case, ostracized participants might show lower accuracies and be slower especially in invalid trials (i.e., when the cue and the target are in an incongruent position). This suggests that they may be more influenced by the social cue (i.e., the gaze) and have difficulties disengaging from it (Hypothesis 3b) [16]. Moreover, in line with Bolling and colleagues [27], we expected age-related differences, with older children possibly being more sensitive to the effects of ostracism on the attentional task. Consequently, we anticipated greater impairment in the accuracies and reaction times for older participants. This might be for different reasons. Indeed, Abrams et al. [24] demonstrated that ostracism threatens the same basic needs in both young and older children and adults, however, in different ways. Older children tend to perceive a greater threat to their need to belong, likely due to the increasing importance of group affiliation at that age. In contrast, younger children experience a stronger threat to their self-esteem. Given that the threat to belonging may drive a stronger motivation to re-affiliate, if ostracism affects children differently based on age, its impact on gaze behavior could also vary [2]. In addition, at these developmental stages gaze processing undergoes important changes [42,46]. Therefore, the effects observed could be driven by these differences in processing eye-gaze, already existing in the population.

We included different SOAs in the gaze cueing task to reduce the predictability of the target by manipulating the time interval between the cue and the target, and because we hypothesized that the effects of ostracism on the gaze cueing response would be more evident at longer SOAs. Indeed, at longer SOAs, children have more time to process the cue and integrate emotional factors, while at shorter SOAs, responses are more reflexive, leaving less time for conscious saccade planning and cue elaboration.

## Method

### Participants

The final sample consisted of forty 6-year-olds (22 boys, M = 6 years 81 days, SD = 122 days), forty 10-year-old children (20 males, M = 10 years 70 days, SD = 158 days) and fifty adults (22 males, M = 24 years and 67 days, SD = 3 years and 7 days). All tested children were enrolled in the 1^st^ or 5^th^ (i.e., last) grade of the primary school. Thirteen additional children and four additional adults were tested but excluded from the gaze cueing task analysis for technical problems (N = 1 adults and N = 4 10-years-old children), because their accuracy in the attentional task was below the accuracy level of 60% (as in [42]) (N = 3 six-years-old children and N = 6 ten-years-old children), or because they already knew the Cyberball game (N = 3 adults).

The final sample for the behavioral coding of Cyberball is slightly different from the one analyzed for the gaze cueing task. Indeed, for the Cyberball, the final analysis comprised thirty-nine 6-year-olds (20 boys; N = 19 ostracized and N = 20 included), fifty 10-year-olds (25 males; N = 25 ostracized and N = 25 included) and fifty adults (22 males; N = 25 ostracized and N = 25 included). This discrepancy between samples is due to the inclusion in the Cyberball behavioral coding sample of children who had the permission for videorecording and completed the Cyberball task, despite being excluded from the gaze cueing task analysis for technical problems or low accuracies. Conversely, some children who did not have the permission for videorecording had to be excluded from the Cyberball behavioral reactivity analysis but could be included in the gaze cueing analyses.

We determined the sample size through a Power Analysis using G * Power Software [47]; a sample size of 27 participants per age group was estimated in order to have 82% probability to detect a significant interaction (α = .05) with a medium effect size (f = .25) with a Within-Between interaction ANOVA, following Cohen’s guidelines [48].

**Fig 1:**
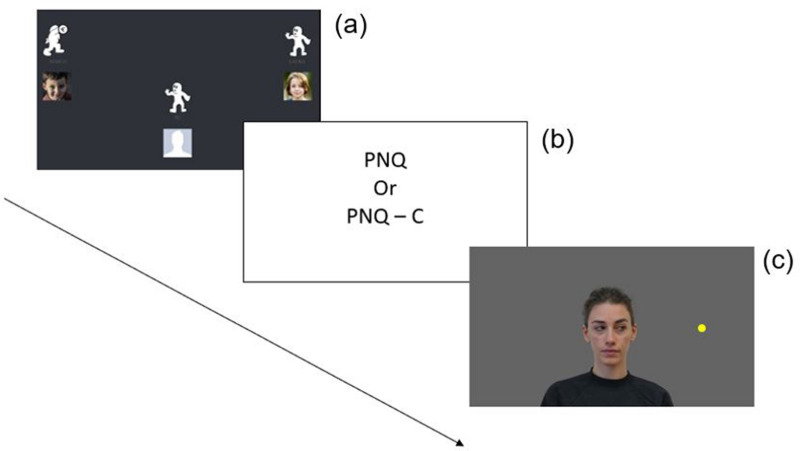
Representation of the three phases of the study. After a brief training phase, participants participated in the actual Cyberball being either included or excluded at the game (a); to make it more credible, participants were told that two other players were playing online.. In the test phase, participants completed the PNQ or PNQ-C (b) and right after played the gaze cueing task being asked to press a key on the keyboard when the target (i.e., colored dot) was presented (i.e., “k” when it appeared on the right and “d” when it appeared on the left side of the face).

**Fig 2:**
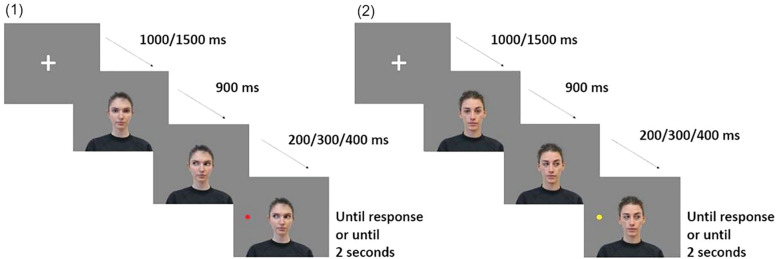
Gaze cueing Task. After 1000/1500ms of fixation cross the face appears and stays with the eyes towards the child for 900ms, subsequently the face gazes right or left and after a SOA of 200/300/400ms, the target appears either in a valid (1) or invalid (2) position compared to the cue.

The protocol was carried out in accordance with the ethical standards of the Declaration of Helsinki (BMJ, 1991; 302:1194) and was approved by the ethical committee of the University of Milano-Bicocca (protocol number: 654). All children were recruited via personal connection with a Comprehensive Institute, based in Sesto San Giovanni (Milan, Northern Italy). Parents, teachers and children were provided with written informing materials about the research project. Adult participants were recruited at the University of Milano-Bicocca or via personal connection. The data collection started on the 13^th^ of April 2022 and ended on the 28^th^ of February 2023. Only children and adults with informed written consent and privacy modules completed and signed were tested. Only children and adults who did not have a neuroatypical development certification were included in the analysis. The individuals pictured in Fig 1 and 2 have provided written informed consent (as outlined in PLOS consent form) to publish their image alongside the manuscript.

### Procedure

All children were tested in their primary school in an individual session. The child was brought into the dedicated testing room, a silent facility provided by the school, and begun the experimental session, which comprised three phases. Children first played the Cyberball game on a computer being either included or ostracized from the game; secondly, they were requested to complete the Primary Needs Questionnaire – Children (PNQ-C; [25]) and, finally, they performed the gaze cueing task (Fig 1). All adult participants were tested in a laboratory of the University. Each session was an individual session, and the phases of the study were the same as for children, with the exception that they were asked to fulfill the adult version of the Primary Needs Questionnaire [12].

After giving a brief explanation of the procedure to the participant, the testing began. To avoid biasing participants, we provided minimal instructions about the Cyberball game before the experiment. Children and adults were told that they were going to play an online ball tossing game with two other participants, sitting in the rooms next door. After each session, participants were debriefed and could ask questions.

#### Cyberball.

The online version of the Cyberball procedure [49] was designed so that the participants’ avatar was at the bottom of the screen and the other two participants were on the up-right and up-left corners of the screen. All children were briefly familiarized with the online Cyberball game (i.e., N = 6 throws) to show them how to play the game and what buttons they had to press on the computer mouse. This training phase was conducted to ensure that all participants understood the task before starting the actual Cyberball game. Participants were informed that these initial throws were part of the training. If they demonstrated understanding of the game mechanics, the procedure could proceed. If any aspect of the game remained unclear, the training phase was repeated until participants fully grasped the rules. The total number of throws during the game was 18 [17,45]. In the inclusion condition, all throws were equally distributed between the three players, while in the ostracism condition the first 6 throws were equally distributed between players and the last 12 throws only between the two avatars. After Quadrelli and colleagues [43], the period of the game was divided as follows to explore behavioral reactions between included and ostracized children: the first six throws of the game were considered a baseline phase since the pattern of throws was the same for both conditions, while the last 12 throws were considered the experimental phase, which differs between conditions. Children were pseudo-randomly assigned to the inclusion or ostracism condition, so that half of the participants were assigned to the inclusion condition and the other half to the ostracism condition.

The adult version of the Cyberball was designed so that labels containing the names of the three participants were positioned underneath the avatars. Also adults were briefly familiarized with the game before the real Cyberball was played. The total number of throws was 30 as in previous studies [15]. Participants were pseudo-randomly assigned to the inclusion or ostracism condition. The first one-third of the throws (N = 10) were considered the baseline phase, as the pattern of throws was the same for both conditions. The remaining 20 throws constituted the experimental phase, which differed between conditions. As was the case for children, in the experimental phase of the inclusion condition adult participants kept on receiving and throwing the ball, while in the ostracism condition they did not receive the ball anymore.

All Cyberball sessions were video-recorded to analyze participants’ behaviors during the inclusion or ostracism conditions.

#### Primary needs threat questionnaires.

As we also wanted to assess whether participants felt their fundamental needs to be threatened, we asked adults and children to respectively fullfill the Primary Needs Questionnaire [12] and the Primary Needs Questionnaire – Children (PNQ-C; [25]). Both questionnaires were administered on a 10.1” tablet and all responses were given by touching or sliding with the finger on the screen. The PNQ and PNQ-C contain questions concerning the four fundamental needs threat (i.e., belonging, control, self-esteem, meaningful existence) and a question regarding the experimental manipulation, an index of how participants felt ignored during the game [12]. Both require a response on a Likert scale ranging from 1 (not at all) to 5 (a lot). Zadro et al. [45] indicated that the PNQ-C is suitable for children aged 8 years and older. Therefore, we implemented the questionnaire on a tablet with visual aids to facilitate comprehension among younger participants, particularly those aged 6 years. While we believe this approach likely improved understanding, further research into the effectiveness of visual aids in enhancing comprehension in younger children would be beneficial.

Finally, we obtained for each participant his/her score for all the four fundamental needs (i.e., the higher the score, the higher the need threat) by making the sum between the scores obtained at the questions related to that specific need [25]. Afterwards, we summed all the scores of each need together obtaining a variable renamed “*Needs Threat*” to achieve a rating of the needs threat for each participant [25]. We also considered the score obtained at the “*Experimental Manipulation”* question.

#### Gaze cueing task.

The stimuli of the gaze cueing task consisted of colored photographs of two white European heritage women actresses posing neutral facial expressions while facing forward and then gazing to the right or left side of the screen. The pictures were taken using a Lumix DMC-FZ300 camera. All pictures were acquired and edited using Photoshop CS6 in our laboratory at the University of Milano-Bicocca. All pictures were cut at half-bust, and the actresses were wearing the same black t-shirt. All pictures were shown against a grey background. The image size was 375 height x 500 width. Stimuli were also equalized for luminance, which did not differ between identities, Kruskal-Wallis H test, χ^2^(1) = 1.19; *p* = .27; ε^2^ = .24, or gaze directions, Kruskal-Wallis H test, χ^2^(2) = 3.71; *p* = .16; ε^2^ = .74.

The stimuli were presented in a pseudo-random order on a 15.6“ portable computer with a 1920x1080 resolution, using the E-Prime software v3.0 (Psychology Software Tools Inc., Pittsburgh, PA). A trial started with a centrally presented fixation cross (duration between 1000 and 1500 ms) and then a face with direct gaze appeared in the center of the screen. After 900 ms the eye gaze moved either to the right or to the left. The target, consisting in colored circles (green, blue, red or yellow) appeared to either the right or the left of the screen, in a spatially congruent (valid) or incongruent (invalid) position with respect to the gaze direction. The Stimulus Onset Asynchrony (SOA) between the eye gaze and the target appearance was set to 200, 300 or 400 milliseconds. Overall, each trial could either be valid (i.e., cue and target in the same direction), invalid (i.e., cue and target in opposite directions) or a catch trial (i.e., no target showing up after the cue). There was a total of 5 blocks, each block was composed of 12 trials (equally divided between valid and invalid trials) and 2 catch trials, for a total of 60 experimental trials and 10 catch trials. The target remained on the screen until the participant’s response [34] or for a maximum of 2 seconds (Fig 2). The prediction of the cue relative to the target’s presentation side was 50% [36]. Between each block there was a pause and participants had to press the spacebar on the keyboard to continue. Participants were asked to make speeded detection of the target by pressing “d” or “k” on the keyboard when the target appeared respectively to the left-side or the right-side of the face. Participants were asked to be as fast and accurate as possible. Responses were allowed after the appearance of the target and reaction times (RTs) were recorded. At the end of each block, the trials (valid/invalid) in which the participant had to respond but did not give an answer were repeated once. The responses to the gaze cueing task were recorded by E-Prime software v3.0 (Psychology Software Tools Inc., Pittsburgh, PA).

#### Affective and behavioral response from off-line coding.

A specific observation tool was created to allow for the microanalytic analysis of facial expressions and posture of children and adults while playing the Cyberball game. The present observation grid was inspired by previous studies [23,43]. Videos were coded by a research assistant who was unaware of the condition of the game, and that proceeded to code facial and postural behaviors that were videorecorded during the Cyberball game. All videos were sub-divided into 2-second intervals in which each behavior was coded as present (1) or absent (0) as in Quadrelli’s research [43]. Our coding categories were divided as follows: (1) Smile (i.e., linked to positive affectivity and composed by: raising of one corner of the mouth, raising both corners of the mouth and/or raising one or both corners showing the teeth), (2) Leaning Forward/Bend Head on the Side (i.e., linked to showing interest and involvement and composed by: leaning forward with the shoulders and/or head towards the computer and/or bending head on the side), (3) Disappointment (i.e., linked to expressing disagreement and composed by: raising the eyebrows, widening the nostrils and/or raising the eyebrows while pulling back the corners of the mouth), (4) Distress Behavior (i.e., linked to expressing anxiety and frustration and composed by: leaning backward, body swaying and adjusting on the chair, pursed/thin lips, clench teeth, tighten jaw and forced swallowing, frowning and looking away from the game). Separately for the baseline and experimental phases, the scores of the different behaviors were summed up and normalized by dividing the final score by the number of expressed behaviors and then multiplied by 100 to obtain a percentage score.

Approximately 30% of the videos (N = 33) were additionally coded in blind by a separate coder unaware of the study hypothesis. Intraclass correlation coefficients (ICCs) were calculated and found to be excellent for all indexes and behaviors in the two phases, with values ranging from .955 to .996 and a mean ICC of .981 (all *ps* < .001).

### Statistical analysis

All statistical analyses were performed on Jamovi 2.3 (https://jamovi.org) using a two-tailed .05 level of significance.

Differences between conditions for children and adults on the manipulation checks (i.e., PNQ and PNQ-C) were tested using independent-sample t-tests. All behavioral reactivity indexes (i.e., Smiles, Leaning Forward/Bend Head on the Side, Disappointment, and Distress Behavior), were analyzed between the three age groups (i.e., 6- and 10-years old children and adults) using an ANOVA with Age and Condition as between-subjects factors and Period (i.e., baseline phase and experimental phase) as within-subjects factor.

For the gaze cueing task, we considered the Reaction Times (RTs), excluding trials where the subjects gave an incorrect answer to the target, and the accuracy of responses. Collected data were analyzed comparing the three age groups using ANOVAs with age and condition (i.e., inclusion vs ostracism) as between factors, and SOA and validity as within factors for each of the measures taken into consideration.

For all analyses when the ANOVAs yielded significant effects, pairwise or independent sample comparisons including ≤ 3 means were performed by applying t tests and Fisher’s least significant difference procedure [48] and Holm–Bonferroni correction was used where appropriate [50]. The data are reported as means and standard deviations (SDs).

## Results

### Primary needs threat questionnaires

A series of independent samples t-test conducted on the PNQ-C results revealed that, for *“Needs Threat”,* ostracized 6-year-old children (M = 19.30, SD = 5.18) did not differ from included children (M = 17.20, SD = 3.32), *t*(38) = 1.53, *p* =.135, *d* = 0.48. Also, at 6 years of age, ostracized (M = 2.35, SD = 1.35) participants did not report to feel more ignored than included ones (M = 1.75, SD = 1.48), *t*(38) = 1.34, *p* =.189, *d* = 0.42. Conversely, ostracized 10-year-olds reported to have felt significantly more their needs threatened (M = 27.60, SD = 6.64) than included ones (M = 17.35, SD = 5.40), *t*(38) = 5.36, *p* < .001, *d* = 1.69, and felt more ignored (M = 3.75, SD = 1.16) than those who were included (M = 1.35, SD = 0.93), *t*(38) = 7.19, *p* < .001, *d* = 2.27. Similar resul*t*s were obtained for adult participants, as ostracized individuals felt more ignored (M = 3.68, SD = 1.22) than included ones (M = 1.40, SD = 0.91), *t*(48) = 7.50, *p* <.001, *d* = 2.12, and reported *t*o feel their needs to be more threatened (M = 40.84, SD = 5.60) than included individuals (M = 28.68, SD = 5.76), *t*(48) = 7.57, *p* < .001, *d* = 2.14.

### Cyberball affective and behavioral response from off-line coding

#### Smile.

A repeated measure ANOVA was conducted to assess whether being included vs. ostracized could affect children and adults’ smiling behavior and whether this was particularly true in the experimental phase, where the throws are equally distributed between players in the inclusion condition and only between the two avatars in the ostracism condition, rather than the baseline phase where all participants receive an equal number of throws. Results showed that there was a main effect of period *F*(1, 133) = 5.49, *p* =.016, η²p =.043 with less expression of smiles in the baseline (M = 7.77%; SD = 18.9%) than the experimental phase (M = 13.3%; SD = 24.2%). There was also a significant interaction between period and condition *F*(1,133) = 14.30, *p* < .001, η²p = .097, with ostracized participants smiling more in the experimental phase (M = 18.7%, SD = 28.8%) than the baseline phase (M = 4.98%, SD = 12.7%), *t*(133) = ‒4.37, *p* < .001. Fur*t*hermore there was a significant interaction between period and age *F*(2,133) = 7.96, *p* < .001, η²p = .107. Post-hoc tests revealed that adults tended to smile more in the experimental (M = 18.8%, SD = 32.06%) than the baseline phase (M = 2.71%, SD = 10.7%), *t*(133) = ‒4.77, *p* < .001. All other comparisons revealed no significant resul*t*s, all *ps* > .08. In addition, there was a significant interaction between condition and age *F*(2,133) = 8.61, *p* < .001, η²p = .115. Post-hoc tests revealed that ostracized 10-year-olds were smiling less (M = 4.90%, SD = 8.06%) than ostracized adults (M = 19.6%, SD = 22.3%), *t*(133) = - 3.17, *p* = .026; and ostracized adults smiling more than included ones (M = 1.88%, SD = 4.81%), *t*(133) = ‒3.82, *p* = .003. Lastly, *t*here was a significant interaction be*t*ween age, condition and period *F*(2,133) = 3.47, *p* = .034, η²p = .050. In order to inspect the significant three-way interaction effect, we conducted three separate ANOVAs taking into consideration each age group separately.

The ANOVA on 6-year-olds revealed no main or interaction effects, all *ps* > .21. Results for 10-year-old participants showed a main effect of condition *F*(1,48) = 4.30, *p* = .044, η²p = .082, with ostracized participants smiling less (M = 4.90%, SD = 8.06%) than included ones (M = 13.1%, SD = 18.1%). Finally, the ANOVA conducted on adult participants revealed a main effect of period *F*(1,48) = 16.2, p < .001, η²p = .253 with adults smiling more in the experimental (M = 18.76%, SD = 32.6%) than the baseline phase (M = 2.71%, SD = 10.7%), and also a main effect of condition *F*(1, 48) = 16.3, *p* < .001, η²p = .238, with ostracized adults smiling more (M = 19.58%, SD = 22.3%) than included ones (M = 1.88%, SD = 4.81%). Finally, a significant interaction effect was found between period and condition *F*(1,48) = 14.4, *p* < .001, η²p = .230. Post-Hoc tests showed that ostracized participants smiled more in the experimental phase (M = 35.16%, SD = 39.5%) than the baseline phase (M = 4.00%, SD = 13.8%), *t*(48) = ‒5.53, *p* < .001, and also smiled more in the experimental phase when compared to included ones (M = 2.36%, SD = 6.86%), *t*(48) = ‒4.09, *p* < .001.

#### Leaning Forward and Bend Head on the Side.

The repeated measures ANOVA conducted on this behavior was aimed to assess whether included participants showed more involvement/interest in the game when compared to ostracized ones. Results revealed no significant main or interaction effects. All *ps* > .059.

#### Disappointment.

A repeated measures ANOVA was conducted to test the hypothesis that, when ostracized, participants express more disappointment as compared to included participants. Results revealed a main effect of condition *F*(1,133) = 6.04, *p* = .015, η²p = .043, with ostracized participants expressing more disappointment (M = 9.30%, SD = 10.96%) than included ones (M = 4.72%, SD = 10.96%). The main effect was further qualified by a significant interaction between period and condition *F*(1,133) = 5.49, *p* = .021, η²p = .040. Subsequent comparisons highlighted that, when ostracized, participants expressed more disappointment in the experimental phase (M = 11.05%, SD = 13.54%) as compared to when included (M = 3.36%, SD = 13.55%), *t*(133) = 3.35, *p* = .006. No other comparison attained statistical significance (all *ps* > .16). In addition, no other main or interaction effect were found, all *ps* > .15.

#### Distress Behavior.

For Distress Behavior we found a significant effect of age *F*(2,133) = 5.02, *p* = .008, η²p = .070. Post-hoc tests revealed that 6-year-old (M = 58.4%, SD = 23.3%), *t*(133) = 2.89, *p* = .013, and 10-year-old children (M = 55.1%, SD = 28.4%), *t*(133) = 2.50, *p* = .028, were expressing more Distress Behavior than adults (M = 41.4%, SD = 29.9%). No other main or interaction effect was found, all *ps* >  0.10.

### Gaze cueing Task

#### Accuracy.

An ANOVA conducted on the accuracies at the gaze cueing task was done to assess whether being either included or ostracized could have an impact on children’s and adult’s accuracy in responding to the targets, and whether there were age differences in how an ostracism experience can impact the performance in the task. We conducted ANOVAs with age and condition as between factors, and SOA and validity as within factors. Results revealed a main effect of validity *F*(1,124) = 12.45, *p* < .001, η²p = .091, with participants being more accurate for valid (M = 0.99, SD = 0.02) rather than invalid (M = 0.98, SD = 0.03) trials. In addition, there was a significant interaction between SOA and validity *F*(2,248) = 4.91, *p* = .008, η²p = .038. Post-Hoc showed that, with a SOA of 400 ms, participants were significantly less accurate in invalid (M = 0.97, SD = 0.06) rather than valid trials (M = 0.99, SD = 0.02), *t*(124) = 4.3, *p* < .001. Analysis also showed a main effec*t* of age *F*(2,124) = 11.22, *p* < .001, η²p = .153, Post-hoc tests revealed that adults were more accurate (M = 0.996, SD = 0.01) than 6-year-olds (M = 0.98, SD = 0.02), *t*(124) = ‒4.12, *p* < .001, and 10-year-olds (M = 0.98, SD = 0.03), *t*(124) = ‒3.93, *p* < .001. Notably, a significan*t* quadruple interac*t*ion was found between age, condition, validity and SOA, *F*(4, 248) = 6.142, *p* < .001, η²p = .090. To explore this interaction, we conducted separate ANOVAs for each age group, maintaining SOA and validity as within-subjects factors and condition as a between-subjects factor.

**Fig 3:**
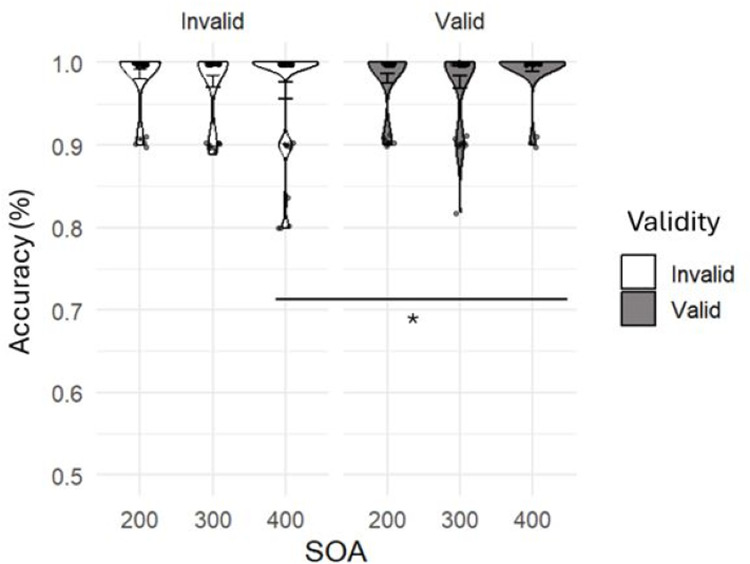
Results regarding 6-year-old children’s accuracy in responding to the target. (*p < .05).

Results for 6-year-old participants revealed a significant interaction between SOA and validity, *F*(2,76) = 3.57, *p* = .033, η²p = .086. A paired sample t-test was conducted to explore such interaction revealing that participants were less accurate in invalid (M = 0.97, SD = 0.06) rather than valid (M = 0.99, SD = 0.03) trials, only when the SOA was 400ms, *t*(39) = 2.94, *p* = .006, *d* = .46 (Fig 3). No other main or interaction effects were found significant, all *ps* > .10.

**Fig 4:**
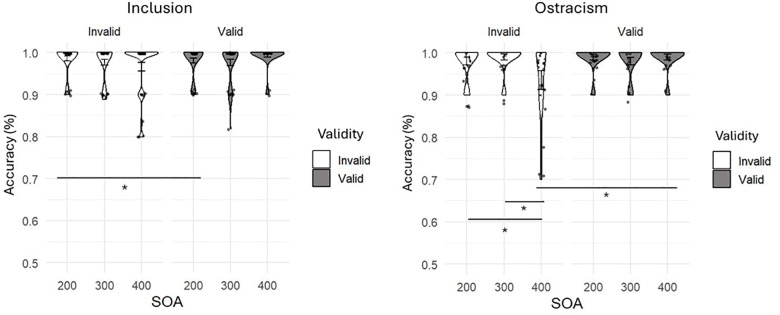
Results regarding 10-year-old children’s accuracy in responding to the target. Considering SOA, validity and condition as independent variables and accuracy as dependent variable (*p < .05). The graphs depict a comparison between valid and invalid trials in the (a) inclusion condition and (b) ostracism condition.

For 10-year-old participants the ANOVA underlined a significant main effect of validity, *F*(1,38) = 6.92, *p* = .012, η²p = .154, with participants being more accurate in valid (M = 0.99, SD = 0.02) as compared to invalid (M = 0.97, SD = 0.04) trials, and also a significant interaction between SOA, validity and condition, *F*(2,76) = 7.41, *p* = .001, η²p = .163. An independent-sample t-test revealed that ostracized participants were less accurate in invalid trials (M = 0.935, SD = 0.099) when compared to included participants (M = 0.99, SD = 0.04), with a SOA of 400ms, *t*(38) = ‒2.12, *p* = .040, *d* = ‒.671. In addition, within the included condition, a paired sample t-test revealed a significant difference between valid (M = 0.995 SD = 0.02) and invalid (M = 0.975, SD = 0.06) trials when the SOA was 200ms, *t*(19) = 2.18, *p* = .042, *d* = .049, while within the ostracism condition a significant difference emerged between valid (M = 0.990, SD = 0.008) and invalid (M = 0.935, SD = 0.099) trials only when the SOA was 400ms, *t*(19) = 2.77, *p* = .012, *d* = .620. Moreover, excluded participants were less accurate in invalid trials when the SOA was 400ms when compared to a 300ms SOA (M = 0.99, SD = 0.03), *t*(19) = 2.77, *p* = .012, *d* = .620, and a 200ms SOA (M = 0.98, SD = 0.04), *t*(19) = 2.27, *p* = .035, *d* = .507 (Fig 4). For adults, all main and intercation effects were not significant, all *ps* > .05.

#### Reaction Times (RTs).

An ANOVA on RTs toward the target was conducted to assess whether being left out from the game could impair ostracized participants’ gaze following. A main effect of SOA was found, *F*(2, 248) = 39,50, *p* < .001, η²p = .242, with participants being slower to respond when the SOA was 200ms (M = 465 ms, SD = 79.47 ms) as compared to a SOA of 300ms (M = 438 ms, SD = 79.58 ms), *t*(124) = 7.37, *p* < .001, and a SOA of 400ms (M = 436 ms, SD = 76.73 ms), *t*(124) = 7.98, *p* < .001. There was also a main effect of validi*t*y, *F*(1, 124) = 183.05, *p* < .001, η²p = .596, as participants were faster for valid (M = 428 ms, SD = 76.05 ms) rather than invalid (M = 465 ms, SD = 76.96 ms) trials. We also found a main effect of condition, *F*(1,124) = 5.44, *p* = .021, η²p = .042, with ostracized participants (M = 462 ms, SD = 74,90 ms) being slower than included ones (M = 431 ms, SD = 74,90 ms), and a main effect of age, *F*(2,124) = 128.85, *p* < .001, η²p = .68. Post-hoc comparisons underlined that adults were faster (M = 325 ms, SD = 83.44 ms) to respond to the target than 10-year-old children (M = 436 ms, SD = 74.63 ms), *t*(124) = 7.06, *p* < .001, and 6-year-old children (M = 578 ms, SD = 74,63 ms), *t*(124) = 15.8, *p* < .001, and that 10-year-olds were faster *t*han 6-year-olds, *t*(124) = ‒8.53, *p* < .001.

**Fig 5:**
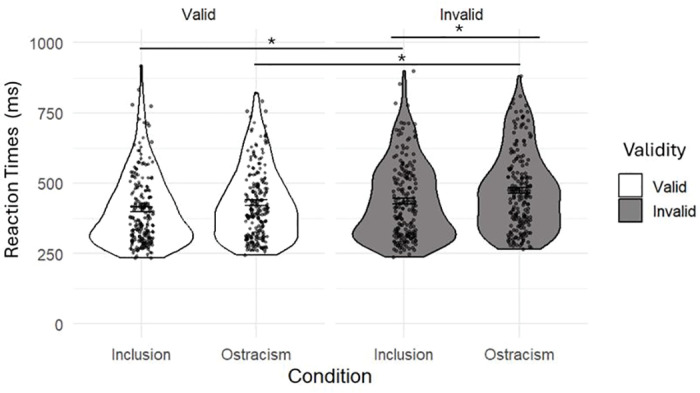
Results regarding adult and children participants’ RTs both in the inclusion and ostracism condition. Considering validity and condition as independent variables and RTs as dependent variable (*p < .05).

Analysis also highlighted an interaction effect between validity and condition, *F*(1,124) = 5.63, *p* = .019, η²p = .043. Post-hoc tests showed that ostracized participants were slower in invalid (M = 483 ms, SD = 76.99 ms) trials rather than valid trials (M = 440 ms, SD = 76.03 ms), *t*(124) = ‒11.24, *p* < .001, and the same occurred for included participants who showed the same pattern when comparing valid (M = 416 ms, SD = 76.03 ms) and invalid trials (M = 446 ms, SD = 76.99 ms), *t*(124) = - 7.89, *p* < .001. Pos*t*-hoc comparisons also underlined a significant difference between invalid trials when comparing the two experimental groups, with ostracized participants being slower to respond than included ones, *t*(124) = 2.75, *p* = .021 (Fig 5). Furthermore, *t*here was also an interaction effect between validity and age, *F*(2,124) = 12.44, *p* < .001, η²p = .167, with adults being faster to respond to valid trials than 10-year-old, *t*(124) = 6.20, *p* < .001, and 6-year-old children, *t*(124) = 14.85, *p* < .001, and 10-year-olds being faster *t*han 6-year-old participan*t*s, *t*(124) = ‒8.21, *p* < .001. Also, in invalid trials adults were faster than 10-year-olds, *t*(124) = 7.61, *p* < .001, and 6-year-old participants, *t*(124) = 16.56, *p* < .001, and 10-year-old being faster than 6-year-old children, *t*(124) = ‒8.50, *p* < .001.

## Discussion

Interpreting eye-gaze has high significance across development [34,36,41]. Research has emphasized that the ability to follow another person’s gaze is crucial in social communication, allowing humans to share attention towards a common argument [34]. Recent studies pointed out that being left out can impact adults’ gaze cueing effect [15], thus modulating explicit response levels and automatic attentional processes. However, how being ostracized affects children’s gaze cueing of attention remains unclear.

The present work aimed at investigating whether and how being ostracized in an online ball-tossing game affects the gaze cueing effect in 6- and 10-year-old children and adults, and their behavioral reactivity during the game. To do so, we implemented a computerized Cyberball game, to which each participant took part, being either included or ostracized. Subsequently, participants were administered the PNQ or PNQ-C and completed a gaze cueing task aimed at investigating the ability to orient their attention to the direction of another’s gaze.

When considering explicit verbal responses collected through questionnaires following the Cyberball game, our study highlights that an ostracism episode threatens fundamental needs in adults and older children. Although 6-year-old children did not show any difference between inclusion and ostracism conditions for the responses to the PNQ-C, 10-year-old children and adults reported to feel their fundamental needs more threatened and felt more ignored when ostracized during the Cyberball game. These results add evidence to the existing literature showing that Cyberball can be an effective experimental tool to manipulate ostracism also at developmental stages, and are also in line with those obtained in the work by Zadro and colleagues [45], where the PNQ-C was deemed suitable for children older than 8-years. Indeed, it is possible that the lack of effects emerging from the PNQ-C for 6-year-old children is due to difficulties in comprehension of the items, as suggested by previous research [45]. Importantly, the coding of behavioral and affective reactions that we conducted on the Cyberball game allowed us to go beyond what can be conveyed by the explicit answers to the questionnaire.

Since the seminal study of Williams and Sommer [44] it was underlined that ostracism has an impact on participant’s behavioral reactivity during the game. Indeed, other studies explored participants’ behavioral and emotional reactions during the Cyberball game obtaining different outcomes [23,43,51]. In our study, we could show that being ostracized vs being included at the Cyberball game differently affects participant’s behavioral reactivity. We considered behaviors possibly signaling positive affect as well as negative affect. For the smiling behavior, 10-year-old children expressed more smiles overall during the inclusion rather than the ostracism condition. As the majority of studies have primarily focused on behaviors indicative of negative affectivity, our findings underscore the significance of incorporating an examination of positive affectivity when considering behavioral reactivity to ostracism vs inclusion events. This broader perspective offers a comprehensive understanding of the impact of Cyberball on participants, across both game conditions. Nevertheless, results on adults seem to go in the opposite direction of older children. Indeed, they smiled more when ostracized and, more specifically, during the experimental rather than the baseline phase. However, as underlined by Williams and Sommer [44], smiling can be considered as an ambiguous behavior. Also, Ekman and Friesen [52] stated that smile should not be considered a single class of behavior, but that it can have many communicatory functions. Indeed, adults tend to smile more when exposed to distressing videos [53] and, when facing ostracism, they expressed more Duchenne (genuine) and non-Duchenne (social) smiling [54]. Thus, it is possible that 10-year-olds’ and adults’ smiles during the game conveyed different communicatory functions. Older children may have smiled to express and communicate enjoyment of being included, while adults’ smiling behavior might be considered as a signal of discomfort due to being ostracized. On the other hand, since it is known that experiencing ostracism is positively related to the acting of prosocial behaviors [44,55], smiling during the ostracism condition for adults could have signaled the attempt to act prosocially to be re-included. This explanation aligns with prior research in adult populations, indicating that individuals exhibit various types of prosocial behaviors when seeking re-inclusion [56].

Furthermore, our work demonstrated that ostracized participants, independently of the age group, expressed more disappointment than included ones, particularly in the experimental phase of the Cyberball, where the pattern of throws differs between the two conditions of the game. This results, allows to infer that all participants felt that they were being left out and excluded from the game, as shown by their behavioral reactions. We did not find any significant difference between included and ostracized players in the expression of distress behavior during the game. This result is in contrast with Mulder and colleagues [51]. The discrepancy might be explained in terms of the measurement used to assess the behaviors indexed. Indeed, Mulder and colleagues [51] considered the duration and intensity of each emotion, employing a micro-coding approach of discrete muscle movements. This method is in contrast to our study and previous research [23,43] which predominantly focused on the presence or absence of the behaviors targeted during the game.

Overall, these behavioral results allow us to observe that, despite the lack of evidence in the PNQ-C for 6-year-old children, both adults’ and children’s behavioral reactivity was affected by being ostracized in the online ball-tossing game. This finding underlies the importance of considering different measures when assessing ostracism processing in developmental samples, combining explicit and involuntary responses.

Moving to the effects of being left out on gaze-following, our results point out how an ostracism experience can influence attention towards eye gaze. Indeed, being excluded from the game affected both accuracy and RTs to the target. When considering accuracy in adults, we did not find any effect, probably because the task was too easy for them, thus determining a ceiling effect. Six-year-old participants showed no significant differences in the accuracy responses between the inclusion and ostracism conditions, which indicates that there is no clear evidence that being left out in an online game had any impact on their accuracy in the gaze-cueing task. However, this result should be interpreted with caution, as several factors may have influenced the outcome. For instance, the PNQ-C may have introduced a delay between the ostracism experience and the gaze-cueing task, potentially diluting the immediate emotional impact of ostracism on task performance. Furthermore, younger participants may have had difficulties comprehending the questionnaire, which could have affected their ability to reflect and report their feelings of exclusion. This cognitive limitation may have contributed to the variability observed in their responses, reducing the likelihood of detecting a clear effect of ostracism. Despite these limitations, we observed that, even at this young age, eye gaze automatically elicited an attentional shift, leading to more accurate responses when the cue and target were congruent [57]. This suggests that while ostracism may not have directly impacted accuracy in this age group, the automaticity of gaze-driven attentional shifts remains robust.

Crucially, accuracy responses in the ostracism vs inclusion condition were highly different for the 10-year-old group. When included, 10-year-old children showed to benefit from the cue with short but not long SOA. Therefore, included older children were fast at modulating their attentional shift relying on the eye cue, but the effect of gaze cueing was lost when they had sufficient time to plan the saccade. Conversely, when 10-year-old children were ostracized, they appeared more susceptible to the influence of gaze cues, especially with extended processing time, leading to a higher number of errors in target response. More precisely, ostracized older children demonstrated to have more difficulties in disengaging from the gaze cue as compared to included ones, resulting in being less accurate when the target did not appear where the gaze was signaling.

Studies with adults demonstrated that, after being left out, participants showed a compensatory desire for renewed social affiliation and thus had more difficulties to disengage from social stimuli signaling a possible re-affiliation [16]. In our study, the averted gaze towards the left or right side of the screen was preceded by an eye contact of 900 ms. This initial mutual gaze can have signaled a potential interest towards the perceiver [58], who felt the possibility of re-affiliation and therefore showed to be highly driven in their responses by the gaze cue. In fact, it is known that seeing another person’s direct gaze elicits positive affective reaction, at the behavioral as well as the electrophysiological level [59,60]. In addition, it has been demonstrated that being ostracized widens the cone of gaze, with ostracized participants reporting a stronger feeling of being looked at by a central face, rather than included individuals [61]. Our data confirms results from available literature, extending these assumptions to school-aged children. In addition, our research expands to school-aged children the finding that the desire to be socially accepted after being left out does not manifest only in explicit behaviors and choices but also in basic, early-stage perceptual processes [16] such as processing someone’s gaze direction [14,15].

Our results on RTs partially parallel available adult literature [15] and extend evidence to children. We were able to demonstrate that ostracism did affect both younger and older children's and adult participants’ gaze cueing of attention. Interestingly, ostracized participants showed to be significantly slower compared to included ones to respond to the target when it was presented in the incongruent position to the gaze cue. This leads us to affirm that ostracism impacts gaze following across development, leading participants to display longer latencies to disengage their attention from the social cue. This was particularly true when the target was in the opposite position of the eyes, in line with what we found for 10-year-old children’s accuracies. Taken together, this evidence underlines how social cues represent an important signal for possible re-affiliation, making it harder for ostracized participants to disengage from them. These results lead to affirm that belonging is a fundamental need [7] and that, when threatened, both children and adults implement a series of changes at the behavioral [12,62] as well as the cognitive level [11,14,15,17] to re-affiliate.

It is also possible that ostracism leads to heightened social attention in general, rather than being solely motivated by the desire for re-affiliation. For example, research by Lyyra and colleagues [61] suggests that ostracism widens the cone of gaze, indicating an increased sensitivity to social cues. Similarly, Gardner et al. [63] found that individuals who experience exclusion demonstrate enhanced attention and memory for social stimuli. According to their social monitoring system model, individuals adaptively respond to threats to their need to belong by paying more attention to relevant social information in their environment. This broader increase in social attention following ostracism could serve a purpose beyond re-affiliation, facilitating adaptive responses to social threats. Additionally, Carter-Sowell et al. [56] showed that ostracism enhances social susceptibility, supporting the idea that exclusion heightens sensitivity to social cues in general. Baumeister et al. [64] also found that social exclusion leads to cognitive impairments, such as reduced speed and accuracy in tasks related to intelligent thought, which may influence baseline reactivity and sensitivity to gaze cues. Therefore, it is plausible that the increased social attention observed in our study is not only driven by re-affiliation needs, but may also reflect a more general adaptive response to social exclusion.

Our findings offer valuable insights into the effects of ostracism on both behavioral reactivity and gaze-cueing across development and in adulthood. Specifically, we observed developmental consistencies in response to ostracism, such as similar expressions of disappointment across ages, while also highlighting notable differences, such as variations in smile expression with age. Furthermore, while certain processes, like reaction times, appear to be similarly impacted by ostracism in both children and adults, other aspects of social stimuli processing show developmental distinctions. These results build on previous research suggesting that children, much like adults, engage in re-affiliation behaviors after experiencing exclusion [16,22,43,65]. This reinforces the hypothesis that the fundamental need for social connection, as well as the mechanisms that drive it, are present early in development, but may manifest differently depending on developmental stages.

Some limitations need to be acknowledged and addressed in future research to ensure a more comprehensive understanding of the phenomena under investigation. Indeed, we did not assess whether individual differences could modulate the behavioral responses during the Cyberball game and the subsequent performance in the gaze cueing task. Tobia and colleagues [26] outlined that self-esteem, popularity, and non-verbal intelligence have a moderating role in the effects of ostracism on children’s cognitive performance. Behavioral and emotional responses to ostracism are heightened in people with low self-esteem [66] and high rejection sensitivity [67] and lowered in people with higher psychological resilience [68]. Future studies might take into consideration individual differences to better understand whether and how they modulate ostracism perception and consequences. Another possible limit of the current study is the use of the same paradigm across a wide age range, from 6-year-olds to adults. Despite this approach offers important vantages, such as the possibility to compare different age-groups for the same measurement, it also presents certain challenges, as younger children may struggle with task comprehension, while adults may experience ceiling effects. Future research should consider adapting the task to better suit specific age groups, adjusting task complexity based on developmental stages.

## Conclusions

Our results demonstrate that an online ball-tossing game can affect the ability to follow another’s gaze cue since childhood, showing that early experiences of ostracism can impact fundamental social cognitive processes from a young age. This highlights the critical role of early social interactions in shaping children’s abilities to interpret and respond to social cues. Processing social stimuli such as eye-gaze is an ability known to be present since birth [32] and is considered particularly relevant because it allows humans to draw inferences about the intentions and future behaviors of others [18], contributing to social and cognitive development (joint attention, [34]; vocabulary acquisition, [69]; social interactions, [70]). From a practical perspective, these findings underscore the importance of early interventions to mitigate the negative effects of ostracism, which could have long-term consequences for social, cognitive development and school participation [71]. Future research may focus on interventions that promote inclusion in school environments to prevent the adverse outcomes associated with social rejection. Ultimately, understanding how ostracism affects children could provide valuable insights to create more supportive and inclusive environments that foster healthy social development.
